# Stigma, Discrimination, and Hate Crimes in Chinese-Speaking World amid Covid-19 Pandemic

**DOI:** 10.1007/s11417-020-09339-8

**Published:** 2021-01-06

**Authors:** Jianhua Xu, Guyu Sun, Wei Cao, Wenyuan Fan, Zhihao Pan, Zhaoyu Yao, Han Li

**Affiliations:** 1 Department of Sociology, Faculty of Social Sciences, University of Macau, Rm. 3001, E21B, Humanity and Social Sciences Building, Av. da Universidade, Taipa, Macau SAR China; 2 Stanley Ho East Asia College, University of Macau, Taipa, China; 3 Chao Kuang Piu College, University of Macau, Taipa, China; 4 Choi Kai Yau College, University of Macau, Taipa, China; 5 Cheng Yu Tung College, University of Macau, Taipa, China

**Keywords:** Covid-19, Stigma, Hate crimes

## Abstract

The Covid-19 pandemic has given rise to stigma, discrimination, and even hate crimes against various populations in the Chinese language–speaking world. Using interview data with victims, online observation, and the data mining of media reports, this paper investigated the changing targets of stigma from the outbreak of Covid-19 to early April 2020 when China had largely contained the first wave of Covid-19 within its border. We found that at the early stage of the pandemic, stigma was inflicted by some non-Hubei Chinese population onto Wuhan and Hubei residents, by some Hong Kong and Taiwan residents onto mainland Chinese, and by some Westerners towards overseas Chinese. With the number of cases outside China surpassing that in China, stigmatization was imposed by some Chinese onto Africans in China. We further explore how various factors, such as the fear of infection, food and mask culture, political ideology, and racism, affected the stigmatization of different victim groups. This study not only improved our understanding of how stigmatization happened in the Chinese-speaking world amid Covid-19 but also contributes to the literature of how sociopolitical factors may affect the production of hate crimes.

## Introduction

At the time of finishing the first draft of this paper (July 16, 2020), the Covid-19 pandemic had led to over 13 million infections and over half a million deaths around the world. Three months later (October 25, 2020), when the paper was revised, the infections and death had increased to 42.6 million and 1.15 million known cases. The Covid-19 pandemic has become the largest disaster for human beings since WWII. As the early epicenter of the pandemic, mainland China has witnessed many cases of Covid-19-related stigmatization targeting various groups of the population. Soon after, stigmatization also spread to other groups in the Chinese language–speaking world, including Hong Kong, Taiwan, and overseas Chinese communities in Western countries. Stigma-incited hate incidents and hate crimes were also reported among oversea Chinese communities.

Using data collected from interviews with stigma victims, online observation, and media reporting, this research identified five stigmatized groups in the Chinese-speaking world, including Wuhan^1^ residents, Hubei residents, mainland Chinese, overseas Chinese, and Africans in China. These five groups may not be exhaustive, but they represent the most frequently affected and discussed population in the early stage of the pandemic. This paper further discusses the reasons behind Covid-19-related stigmatization. It was found that while fear of infection was the common denominator for all the stigmatization displayed towards those different groups, specific reasons such as food and mask culture, political ideology, and racism played different roles for different targeted groups. This research not only provided a typological analysis of the victim groups of Covid-19-related stigmatization but also comprehensively evaluates the various reasons behind the stigmatization, which may shed light on how to reduce such disease-related stigmatization in the future. It should be noted that since the pandemic is still going on, the research findings are far from exhaustive or definitive. However, it should provide a basis for later research based on more complete and comprehensive data.

## Disease-Related Stigma, Discrimination, and Hate Crimes

Stigma has long been a topic addressed in sociology and criminology (Goffman 2009; Herek 2008). The research on stigma-related infectious disease has also become a significant branch under this broad topic. Much research has explored how patients of infectious diseases such as leprosy, AIDs, tuberculosis, SARS, and Ebola faced stigma in various societies (Gee and Skovdal 2018; Des Jarlais et al. 2006; Kleinman and Watson 2006; Lee 2014; Mak et al. 2006). Apart from patients, disease-related groups and communities may also be stigmatized. For example, medical and public health workers who battled against infectious diseases may be discriminated against by their local communities (Gee and Skovdal 2018; Ramaci et al. 2020b). Some specific communities may also be stigmatized and discriminated due to the believed origins of the infectious diseases. For instance, Russian Jewish immigrants from Eastern Europe were blamed for the outbreaks of 1892 typhus and cholera in the New York city in the USA; the Chinatown community were blamed for the outbreak of the bubonic plague in 1900 in San Francisco and Native Americans for the outbreak of 1993 Hantavirus in the USA, and Asian communities were stigmatized for the outbreak of the 2003 SARS epidemic in some Western countries (Person et al. 2004; Bruns et al. 2020). The consequence of stigmatization could be severe. Patients may delay their seeking for medical service and treatment (Person et al. 2004; Wynne et al. 2014). Victims may also be denied access to job markets and public services such as schooling (Lee et al. 2005). They may also be excluded from interpersonal relationships and suffer from mental health problems (Ramaci et al. 2020a). In particular, the stigmatized population has long been found to have high risks of becoming the victims of hate crimes (Bruns et al. 2020; Herek 2008; Niang et al. 2003; Sanders 2016).

During the Covid-19 pandemic, hate incidents and crimes were widely reported. In the USA, the FBI warned about the increase in hate crimes against Chinese people (ABC March 27, 2020). It was reported that during the 8 weeks from March 19, 2020, to May 13, 2020, 1843 cases of hate incidents and hate crimes against Chinese and Asians were reported to the Stop AAPI Hate Reporting Center in the USA. These cases included verbal harassment (69.3%) and shunning (22.4%), physical assaults (8.1%) and spitting or coughing (6.6%) as well as other civil rights violations, such as workplace discrimination (4.8%), being barred from establishments (2.9%), and being barred from transportation (1.1%) (Stop AAPI Hate Reporting Center 2020). In the UK, it was reported that anti-Asian hate crimes had risen by 21% since the pandemic started until May 2020 (The Guardian May 13, 2020), although assessing the exact number of such crimes was always problematic. Another piece of research reported that the probability of being a victim of hate crimes after the appearance of Covid-19 for ethnic Chinese in London increased from around 3% in February 2020 to over 16% in March 2020 while there was no significant increase in hate crimes against other ethnic groups in the same period (Gray and Hansen 2020). In Italy, the civil society group Lunaria collected over 50 cases of assaults, bullying, and discrimination against Asians from February to early May 2020 (Human Rights Watch May 15, 2020). In China, xenophobia emerged and foreigners and Africans in particular experienced verbal violence, being shouted at and scolded as “foreign trash” (Guardian March 29, 2020). Therefore, it is of some interest to understand how the evolution and character of stigma and hate incidents and hate crimes occurred during what was evidently a criminogenic time.

## Research Questions

At the beginning of the pandemic in January 2020, various groups of the population experienced stigma in mainland China where the pandemic started. When the coronavirus spread out from China, some overseas Chinese also faced stigma in their host countries. In March 2020, when the first wave of the pandemic had largely been contained through the government’s drastic methods of lockdown, China started to face the risk of imported cases. Some foreigners in China (particularly Africans in Guangzhou) faced severe stigma and discrimination. In this paper, we will explore how different groups became the victims of stigma and even hate crimes in the Chinese language–speaking world at the early stage of pandemic, mainly from late December 2019 to early April 2020.

Two clarifications should be made before we present our research questions. On the one hand, we use the term Chinese-speaking world to include both the population of Chinese in Greater China (that is, mainland China, Hong Kong, Macau, and Taiwan) and overseas Chinese. On the other hand, we set our research time frame from the outbreak to early April for two main reasons. Firstly, although there was no clear-cut point, the stigmatization of some groups, although it may still persist today, started to fade off after Covid-19 became a global pandemic. Discrimination against Wuhan and Hubei residents withered once the coronavirus spread all over China. Discrimination towards mainland Chinese in Hong Kong and Taiwan, and overseas Chinese in Western countries also started to fade when locally transmitted cases exceeded the early imported cases. Secondly, since the pandemic is still going on, setting a time frame makes the data collection and analysis process more feasible. Otherwise, the process would be endless.

The following two research questions are specifically addressed: (1) which groups became the victims of stigma and hate crimes during the outbreak of Covid-19 pandemic in the Chinese-speaking world? And the answer was that five targeted groups could be identified. Although that list may not be completely exhaustive, it necessarily contained the most significant cases. This answer led to our second question: (2) What factors contributed to the stigmatization and even hate crimes directed at those groups? The exploration of these questions may shed light on possible solutions to reduce disease-incited stigma and hate crimes in future.

## Data and Methods

Multiple strategies were employed in data collection, including interviews with victims, online observation, and media content analysis. The first set of data came from interviews with the victims themselves, and they were recruited through the research team’s^2^ network with snow-ball sampling strategies. Since Wuhan residents were the first and most obvious group of those stigmatized, our interviewees were heavily drawn from Wuhan residents. Altogether, 31 interviewees who left Wuhan shortly before the lockdown from Macau SAR, Shanghai, and 12 other provinces including Gansu, Guizhou, Hainan, Hebei, Henan, Hunan, Jiangsu, Jiangxi, Shandong, Liaoning, Yunnan, and Zhejiang were interviewed. All these interviewees left Wuhan shortly before the draconian 76-day lockdown of the city started on January 23, 2020. Since overseas Chinese were the largest group who suffered from hate incidents and crimes, nine overseas Chinese were interviewed about their observations of the phenomenon. Overseas Chinese from the USA (4), the UK (2), Australia (1), Netherlands (1), and South Korea (1) were also recruited through the research team’s network. Altogether, 40 interviews were conducted in March and April in 2020, and subjects were mainly asked about their experience and their observation of being discriminated due to the coronavirus and how they responded to stigma if any.

The second set of data came from online observation between January and April 2020. From the very beginning of the outbreak of the pandemic, close attention was paid to the discussion about stigma and hate crimes on various social media such as Weibo (微博), WeChat Moments (微信朋友圈), Douban (豆瓣), Twitter, Instagram, and Facebook. We particularly collected articles that had been posted on the topic in “hotly searched” (热搜), the most discussed daily issue on Weibo. We also used keywords “pneumonia and discrimination” (肺炎 + 歧视) to search in Weibo. Thirty-two posts and their comments were collected for analysis. In particular, all seven research team members were active WeChat users. We kept a close eye on WeChat Moments as well as WeChat groups, the most popular social media in China in which users shared their thinking or re-posted others’ writings, on a daily basis to monitor the discussion of discrimination and stigma due to the pandemic.

The third set of data came from data mining in Wisers (慧科), one of the largest Chinese newspaper databases composed of around 1500 newspapers published in mainland China, Hong Kong, Macau, and Taiwan and overseas Chinese communities. The keywords “pneumonia and discrimination” were used to search in the database. A total number of 2198 newspaper articles published between December 8, 2019, and March 31, 2020, were identified. These articles were downloaded and read manually to summarize relevant discriminatory events and reports. Other variables such as news agencies, publishing dates, and news titles were also coded for analysis.

The fourth set of data came from news reports from mainstream international media. The research team searched reports related to stigma, discrimination, or hate incidents/crimes due to Covid-19 in CNN, BBC, and *New York Times*, *Financial Times*, and the *Guardian*. Ninety-seven news reports which specifically addressed the issue were collected for analysis.

## Victim Populations of Stigma and Hate Crimes amid Covid-19

**Fig. 1:**
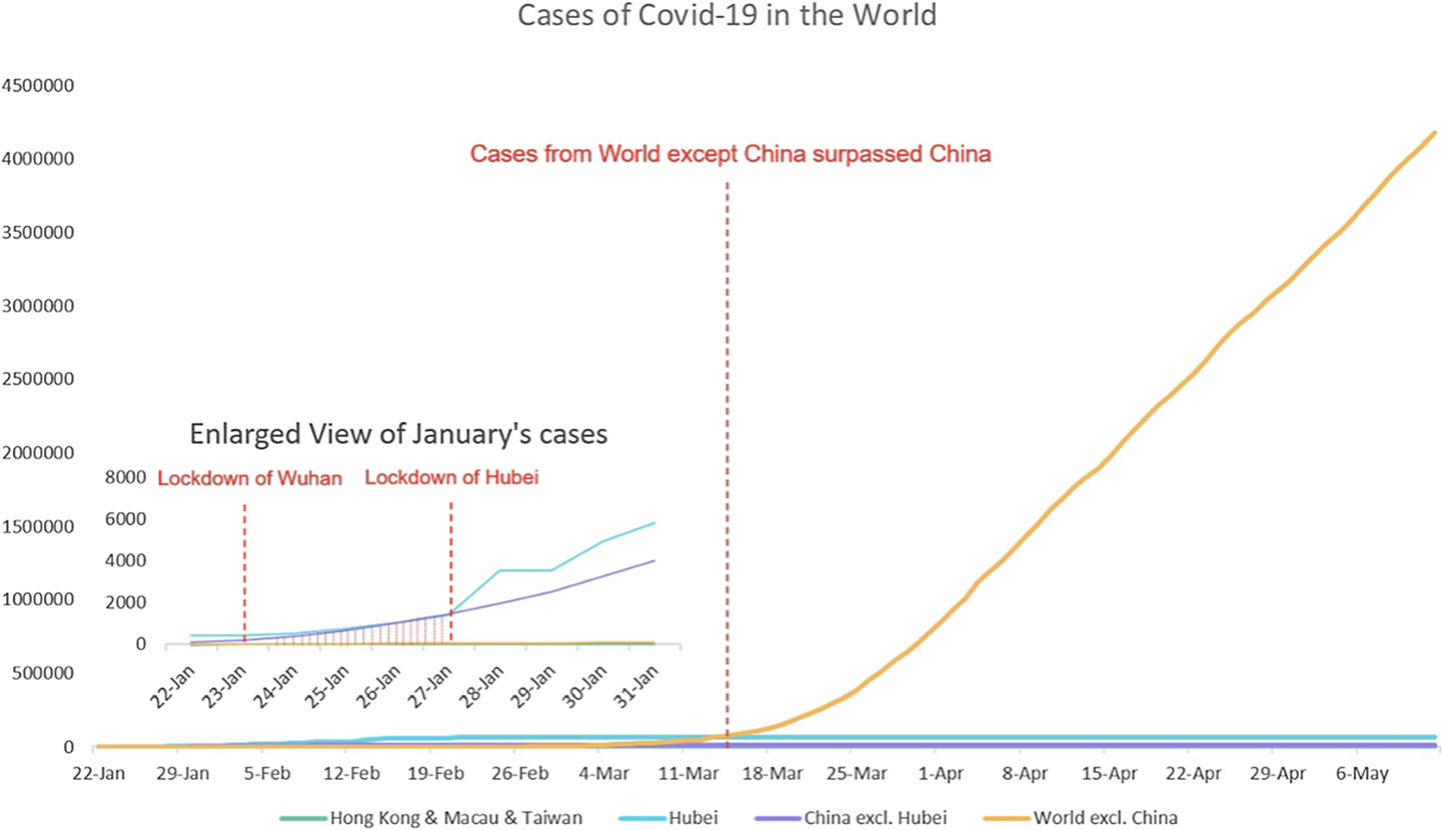
Total confirmed Covid-19 cases from January 22 to May 6, 2020, of Hubei, Hong Kong and Macau and Taiwan, China excluding Hubei, and World excluding China. (Data source: Johns Hopkins University Center for Systems Science and Engineering, 2020)

The situation of Covid-19 changed dramatically since its early outbreak (Fig. 1). At the very beginning, the world’s Covid-19 confirmed cases were concentrated in China, while the China’s cases were themselves concentrated in Hubei province. It was not until mid-March that cases outside China surpassed those in China itself and embarked on their explosive growth after China had largely controlled the first wave of its coronavirus in April. By early May, the cases outside China had surpassed by 50 times those inside China and kept growing exponentially.

The ever-changing situation of Covid-19 has led to the stigmatization to several targeted victim groups in the Chinese-speaking world, including Wuhan residents, Hubei residents, mainland Chinese, overseas Chinese, and Africans in China.

### Wuhan Residents

On the last day of December 2019, China Central Television, the official mouthpiece of the party-state, reported the first several cases of what was called “pneumonia of unknown cause in Wuhan”. It was reported that all the patients were from Wuhan, with the majority related to the Wuhan Southern China Seafood Market (CCTV News December 31, 2019). The market was then reported to be closed the next day for subsequent disinfection. Such news released in the official media left the public with the impression that the pneumonia was linked to Wuhan residents, and the initial term “pneumonia of unknown cause in Wuhan” further strengthened that impression.

Since then, the Wuhan government started to report the confirmed cases. After long denials, the Chinese government finally confirmed the path of human-to-human infection and revealed that wild animals could be the source of the virus on 20 January (Xinhua January 21, 2020b). Six days later, Chinese official media confirmed that a large quantity of coronavirus cases could be traced to the Wuhan Southern China Seafood Market where wild animals were sold (Xinhua January 27, 2020c). The report reminded the Chinese population of the 2003 SARS, which also originated from wild animals. Since then, public fear and anger towards Wuhan residents started to emerge in China. On 23 January 2020, 1 day before the Chinese New Year, an unprecedented measure was taken by the Chinese government to lockdown the whole city of Wuhan. Eleven million Wuhan residents were barred from leaving not only the city but also their residential compound. The drastic lockdown sent a strong signal that the virus could be extremely contagious and out of control. However, it was reported that nearly five million Wuhan residents had left the city shortly before the lockdown mainly because of the Chinese New Year travel rush (CGTN January 27, 2020b). The highly mysterious nature of the coronavirus and the large number of outgoing people from Wuhan led to the exercise of severe vigilance over, and the stigmatization of, Wuhan residents in China. Our interviews with returnees from Wuhan revealed that many of them faced discrimination. Some reported that their private information—including that contained on their ID card and their telephone numbers—was disclosed in the local WeChat group. A university student from Wuhan who returned to Handan, Hebei province, worried the disclosure of such private information and said:

“One of my schoolmates saw an excel file in the local WeChat group, which contained my name, age, gender, ID card and telephone numbers and address. My mother’s classmates also saw this file.”

Some Wuhan returnees became victims of cyberbullying after the disclosure of their personal information. Their neighborhoods called them or found them on WeChat to abuse them verbally. One interviewee remarked that his schoolmates (who also returned home from Wuhan) “were contacted by strangers through phone calls and text messages with offending words such as ‘you should die in Wuhan alone!’.”

Other interviewees complained about the government’s policy of enforcing an unnecessary mandatory quarantine. One Wuhan returnee in Yunnan complained that he was twice tested negative for coronavirus and quarantined in a hospital for 2 weeks before being allowed to return home. However, even so, he was still forced by the local government to stay in quarantine for another 2 weeks. Another interviewee said:

“I saw that one of my classmates (who returned to Xinjiang from Wuhan) complained in WeChat Moments that he was forced to do the coronavirus test even though he had left Wuhan two months ago. I think this is really unnecessary.”

### Hubei Residents

**Fig. 2:**
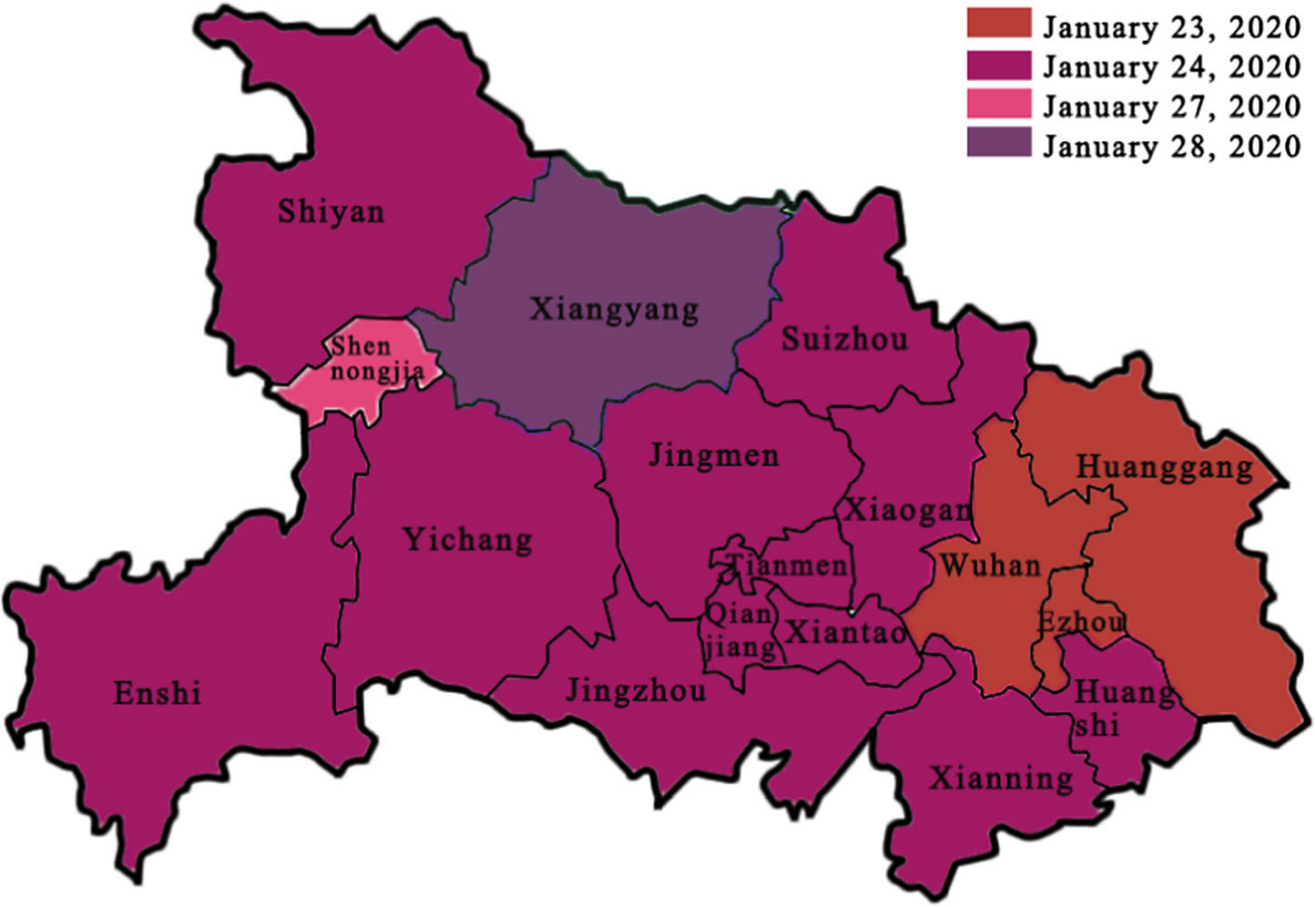
The time of lockdown of all 17 cities in Hubei province

Wuhan, as the capital city of Hubei province, attracted a large number of intra-provincial migrant workers. The migration data from Baidu Map showed that from the start of the Spring Festival travel rush to the lockdown (January 10–22, 2020), nearly 70% of those who left Wuhan went to other cities in Hubei province (CGTN January 27, 2020a).The travel rush posed an immediate risk of the spread of the virus to other cities. After Wuhan was locked down at 10 a.m. on January 23, other cities in Hubei province quickly followed. From the earliest case in Ezhou, which sealed the city at 11:20 a.m. on January 23 to that of Xiangyang on January 28, 17 major cities in Hubei were all locked down (Fig. 2). The dramatic shutdown sent out a strong message that Hubei province was in a dire situation. As shown in Fig. 1, since January 23, the confirmed cases in Hubei grew at a tremendous rate, accounting for most of the cases in China.

**Fig. 3:**
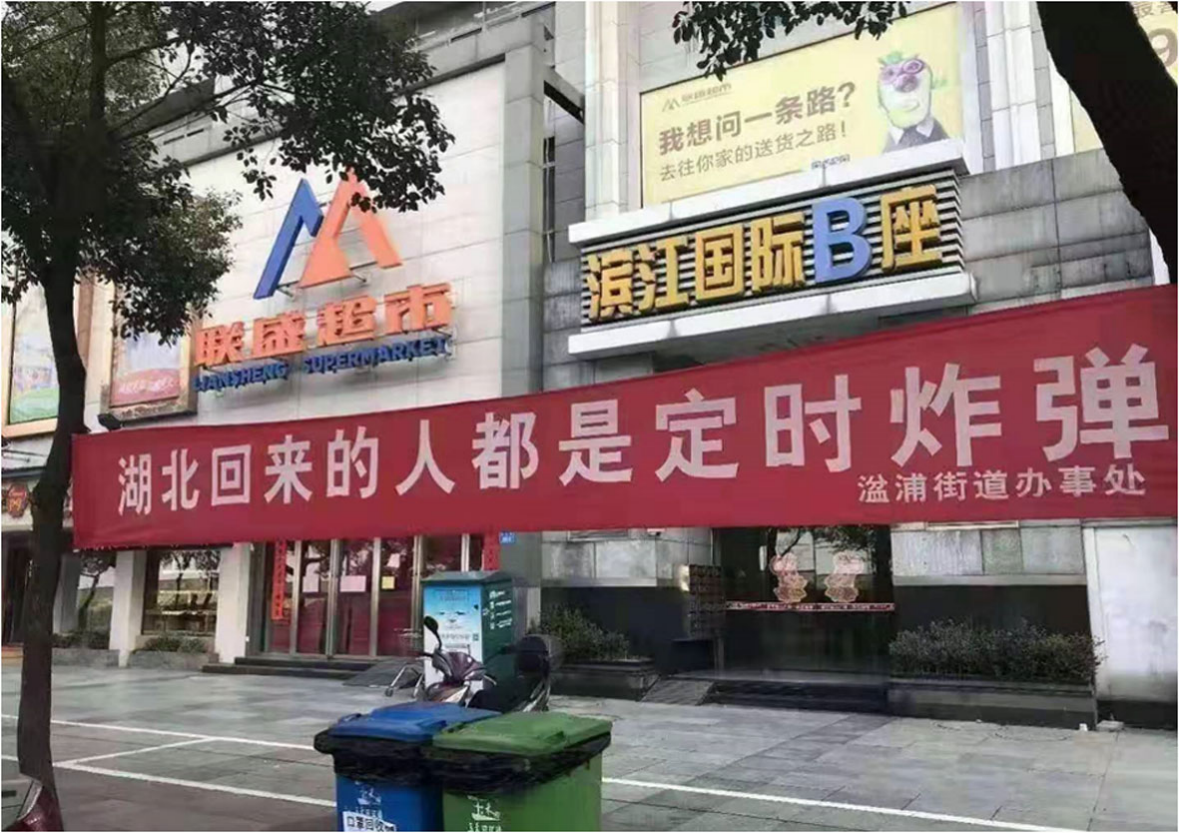
A banner made by a local government (Street Office) in Jiujiang city, Jiangxi province, reads “All returnees from Hubei are time bombs”

With the lockdown of cities in Hubei and the spread of the pandemic, the primary victims of discrimination gradually expanded from Wuhan residents to Hubei residents. People in other provinces became very wary of those who returned from Hubei. A discriminatory street banner from Jiangxi province reminded residents that “all returnees from Hubei are time bombs” (Fig. 3).

Drivers whose vehicles had a Hubei license faced severe stigma. In China, the first character of a vehicle license plate is a Chinese word denoting different provinces or regions, allowing people to quickly identify the provenance of a vehicle. Since “*e* (*鄂*)” represents Hubei, after the lockdown of the whole Hubei province, the appearance of vehicles with an “*e* (*鄂*)” license outside Hubei caused great panic and discrimination. Vehicles with Hubei license plates were officially denied entry to all other provinces, regardless of whether their occupants were infected with Covid-19. Such a policy became a nightmare for many Hubei vehicle owners. Some were stuck on the highway as various local governments did not allow them to get off. For example, Mr. Xiao, a truck driver from Tianmen, Hubei, set out from Hubei on January 7 to transport goods through Yiwu in Zhejiang province, Shenzhen in Guangdong province, and Fuzhou in Fujian province, and then arrived in Dazhou in Sichuan province on January 24. At this time, the pandemic situation in Hubei had turned severe and the prevention measures in place in other regions were stringent. After unloading his cargo in Dazhou, Xiao was reported by the local people to the police because of his Hubei license. Even though Xiao presented his nearly-20-day highway toll receipts as an alibi, and had normal body temperature and negative test results, he was still not allowed to stay in Dazhou and was urged to leave as soon as possible. Since the highway leading back to Hubei had also been closed, Xiao was not able to go home. In addition, other regions also refused to let him get off the highway because of his Hubei license plate. Xiao could only rove on the highway, from Sichuan to Shaanxi. Along the way, he could only take a break in the emergency lanes, and was so tired that he nearly ran into the roadside barrier on several occasions. Eventually, with the help of traffic police in Hanzhong, Shaanxi, he was able to stay in the city’s highway service area for another 48 days before returning (WSJ March 10, 2020; Chinese Business View March 18, 2020). In another case, Mr. Liu, a Hunan native who worked in Wuhan with a Hubei licensed car, began a trip from Hubei to Fujian, Guangxi and Yunnan in mid-January. As the outbreak worsened, he could not get off the highway but had to park his car in a highway service area and slept in the car. Later he was tested negative for the virus and finally found a hotel in Guangxi 2 days later. However, the local police asked him to do the test again regardless of his having been tested negative. Liu had to return to the highway service area again (Hongxing News January 28, 2020). In the above cases, both Mr. Xiao and Mr. Liu literally became like the condemned Flying Dutchman, the captain of the legendary ghost ship that could never make port and was doomed to sail the oceans forever (Bonner 1946).

**Fig. 4:**
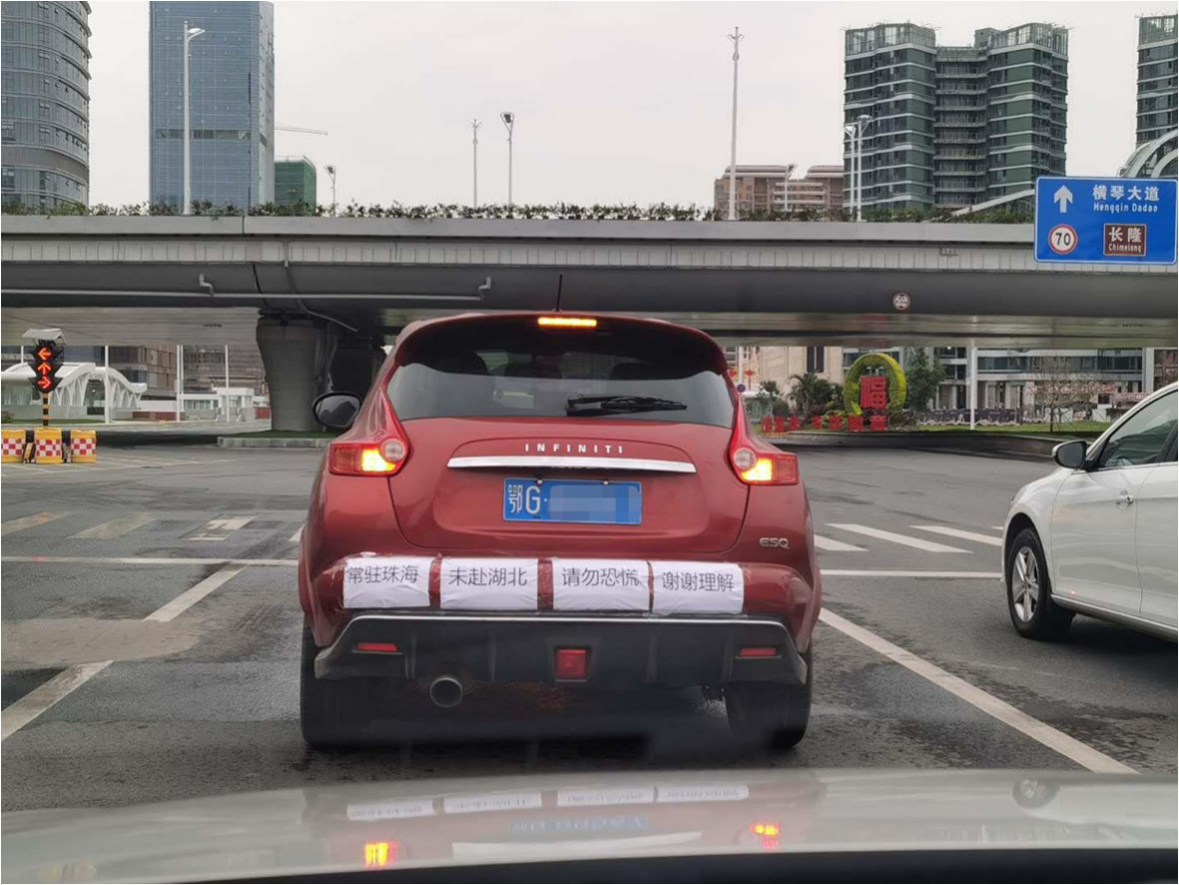
A car owner in Zhuhai, Guangdong province, posted a notice at the back of car. It read “The car owner has long been living in Zhuhai, not visited Hubei. Please don’t panic. Thanks for your understanding”

The discrimination against Hubei citizens was further evidenced by the fact that some residents in other provinces reported vehicles bearing Hubei plates to their local governments, the police, or the hotline for emergency medical service in China. Such a response frustrated Hubei car owners as some of them had not been to Hubei for months or even years. To deal with such stigmata, some of them posted notices on their cars, claiming that they had not been to Hubei for a long time (Fig. 4).

After the lockdown in Hubei was officially lifted, Hubei vehicles were still prevented from entering some provinces. On March 27, a rare violent confrontation occurred between two police forces from Hubei and its neighboring province Jiangxi when the police from Jiangxi prevented the entry of Hubei vehicles. In the conflict, tens of police from each side attacked each other violently. Hundreds of citizens also joined the conflict and several police vans were overturned (Global Times March 27, 2020).

**Fig. 5:**
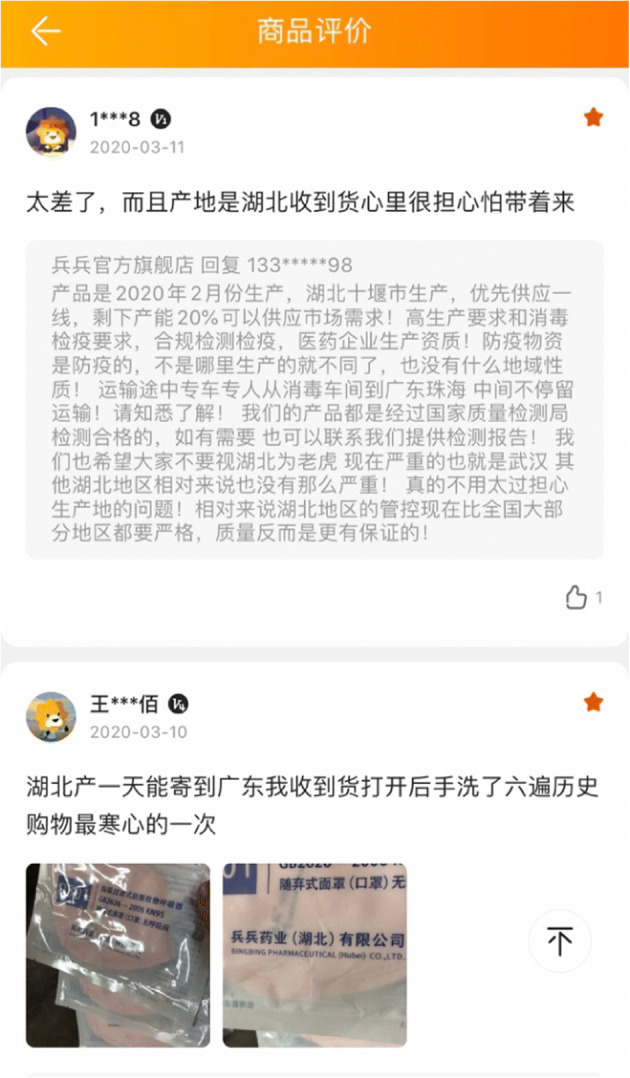
Two online shopping comments on products from Hubei. The first comment reads: “It is terrible. The place of production is Hubei. I was afraid that the product could bring the virus to me after I received the product.” The second reads: “The product from Hubei could be delivered to Guangdong in one day. I washed hands six times after I opened the express box. This was my worst shopping experience”

Besides discrimination against Hubei residents, products made in Hubei were also stigmatized. Some customers made discriminatory comments about the goods produced in Hubei in the online shopping platform Suning.com (see Fig. 5). One said he/she was afraid that the products could contain the virus; another said that he/she washed their hands six times after knowing the products were from Hubei.

Some Hubei ID card holders also suffered from stigmatization. The question “how much discrimination are Hubei citizens now facing outside Hubei?” was posed in Zhihu (知乎, a popular Chinese website for questions), and it attracted much attention. Two commonly shared experiences of stigma were those of not being allowed to stay in hotels and of being evicted by landlords due to their Hubei ID cards. One comment sarcastically remarked that: “It turns out that Covid-19 could be transmitted through ID cards.” As the pandemic was gradually controlled in China and people began to resume work in April 2020, employment discrimination against Hubei ID holders began to surface. Many companies refused to hire or fired Hubei employees without offering a valid reason. All this discrimination towards Hubei citizens was severe enough to attract official attention. *The People’s Daily*, another mouthpiece of the Chinese party-state, said that on the way back to work, we should not “freeze Hubei citizens’ hearts” by discriminating against them (People’s Daily March 20, 2020). On April 23, the Supreme People’s Court (SPC) highlighted that it would address the issue of employment discrimination against Hubei workers (Xinhua April 24, 2020a).

### Mainlanders

The third group suffering discrimination was mainland Chinese in Hong Kong and Taiwan. As Fig. 5 shows, Taiwan began to screen passengers from Wuhan from the end of December, while the Hong Kong government held several meetings to prepare schemes to prevent the coronavirus spreading in early January. Both Hong Kong and Taiwan paid close attention to the mysterious virus long before the Chinese government officially reported it to WHO (the World Health Organisation) on January 3, 2020. The lockdown of Wuhan on January 23 sent out a strong signal that the situation was too severe to control. As the pandemic spread, cases in mainland China skyrocketed after January 27, and the cumulative number of confirmed cases reached 11,791 by the end of January. However, as shown in Fig. 1, the confirmed cases in Taiwan and Hong Kong were much lower compared to those in the mainland. In this context, mainlanders were regarded as threats and being discriminated in Hong Kong and Taiwan.

Stigmatization existed both in physical space and cyberspace. In daily life, it was meted out towards mainlanders in public places such as restaurants. For example, some restaurants in Hong Kong refused to serve people who spoke Mandarin rather than the local Cantonese. An article in the *South China Morning Post*, a Hong Kong local newspaper, reported on March 5 that:

“There were nearly one hundred restaurants that used anti-disease as an excuse to refuse serving travelers from mainland China. For example, some eateries posted messages online or displayed notices at their premises that ‘due to the difficult situation of the outbreak, mainlanders will not be served.’ The post also especially pointed out that ‘those who pay no attention to personal hygiene and speak Mandarin (except people from Taiwan), please leave.’ The best illustration of this point might be, a consumer was required to show a Hong Kong identity card after speaking in Mandarin to ask a table.” (South China Morning Post March 5, 2020)

**Fig. 6:**
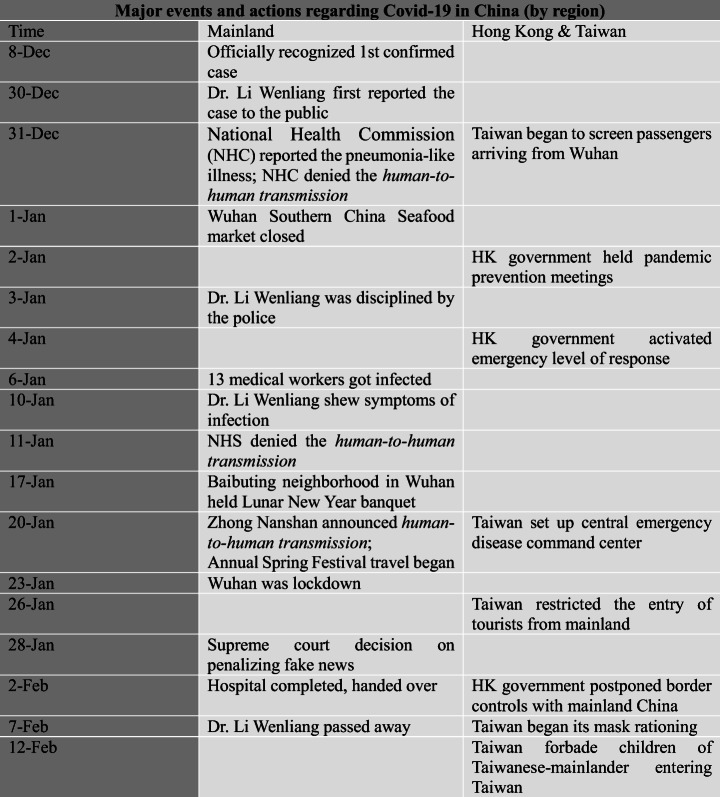
Timeline of major events and measures regarding Covid-19 in mainland China, Hong Kong, and Taiwan

In the cyberspace, mainlanders were depicted as rude, uncivilized outsiders who should be held responsible for the problems caused by the outbreak. In Taiwan, the authority prohibited the mainland spouses and children of Taiwanese citizens from entering on February 12 (Fig. 6).

### Overseas Chinese and Asians

With the coronavirus spreading globally, some overseas Chinese and Asians started to become victims of stigma and even hate crimes. Some faced avoidance in public spaces. For instance, Asian supermarket owners in Cologne reported that there was a phenomenon of “Asian face detours” in which people would avoid coming close to Asians in Frankfurt (China News February 12, 2020c). Others also faced a decline in the provision of medical services and verbal accusations such as “go back to Asia” and “take the virus back” (News April 2, 2020). Stigma and discrimination could also turn to violence. In early February, North Yorkshire Police in the UK probed four racist coronavirus-related hate crimes in which victims were verbally abused in early February (BBC February 6, 2020b). A Chinese man living in Turin in Italy for many years was beaten by two Italians who talked about “getting the virus out of Turin” (Lastampa February 12, 2020). In particular, hate incidents and hate crimes were widely reported in the USA and other parts of the world from March onwards. For instance, a teenage boy in Los Angeles County was beaten up by bullies who accused him of having the coronavirus (CNN March 21, 2020f); a 2-year-old and a 6-year-old were stabbed in a Texas Sam’s Club as the perpetrator thought the family was Chinese and spreading the disease; a 47-year-old Asian man in Queens, New York City, was harassed and pushed by a 44-year-old man; the writer Jeff Yang, co-host of a podcast about being Asian in America and a frequent contributor to CNN Opinion, faced racist aggression when a woman shouted profanities at him and coughed in his direction while shopping in Los Angeles; a 51-year-old woman on a New York City bus was verbally abused and accused for causing the coronavirus by teens; a 59-year-old man was kicked by a 13-year-old boy in New York City (CNN April 11, 2020b).

Not only Chinese but also the whole Asian community faced increased stigma. The data collected by the Asian Pacific Policy & Planning Council revealed that out of 1843 reported hate incidents and hate crimes due to Covid-19 in the USA in March and April, nearly 60% of victims were non-Chinese Asians (Borja et al. 2020). The following examples were just the tip of an iceberg of such discriminatory experiences: an Asian woman in New York city was hit on the head with an umbrella and asked where her mask was (CNN April 6, 2020a); a group of teenagers attacked a young Singaporean man in London, punching and kicking him while shouting about the coronavirus; an Asian woman walked into a park and a group of mothers screamed for their kids to get away from her; a middle-aged Asian woman wearing a mask was going for a walk when a woman shouted at her to get away from her; a man spat at an Asian man waiting for the subway; a man spat at an Asian woman walking to her gym; a woman refused a coffee from a barista because she thought the barista was Chinese; a teenage boy kicked a 59-year-old Asian man in the back; a man chased an elderly Asian woman down the street in Miami, Florida, with a bottle sanitizer to clean “her ass”; a woman punched a young Asian woman in the subway, possibly dislocating her jaw (NYTimes April 12, 2020). Although the abovementioned individual cases could be anecdotal, other systematic research did confirm the rise of hate crimes against oversea Chinese and Asians (Gray and Hansen 2020).

### Africans in China

**Fig. 7:**
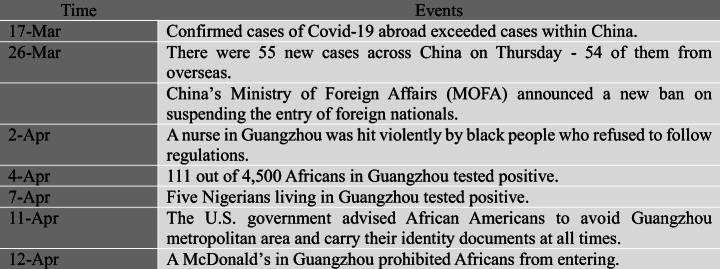
Timeline showing some important events related to stigmatization against foreigners in China

When the number of confirmed cases outside China exceeded that in China on March 17 (Fig. 7), China started to face the risk of receiving imported cases. On March 26, there were 55 new cases across China and 54 of them were from overseas (BBC April 17, 2020a).

**Fig. 8:**
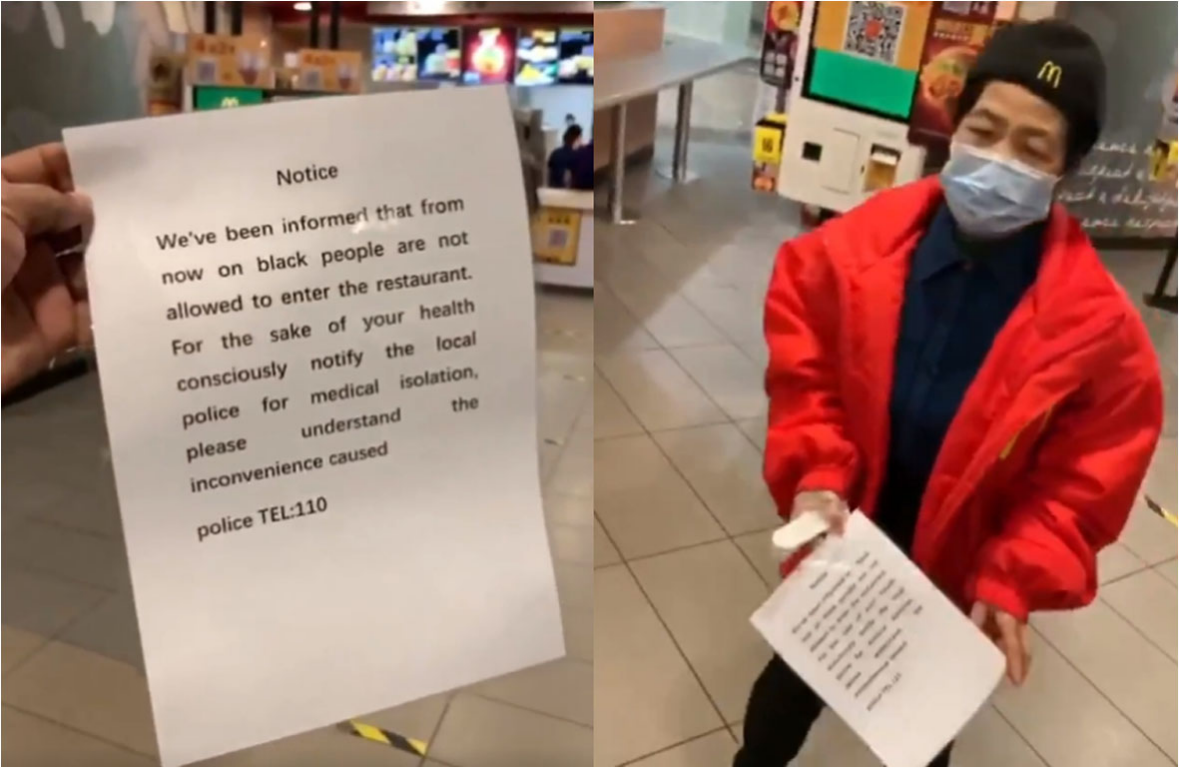
A sign barring Africans from entering a McDonald’s in Guangzhou, China, April 12

**Fig. 9:**
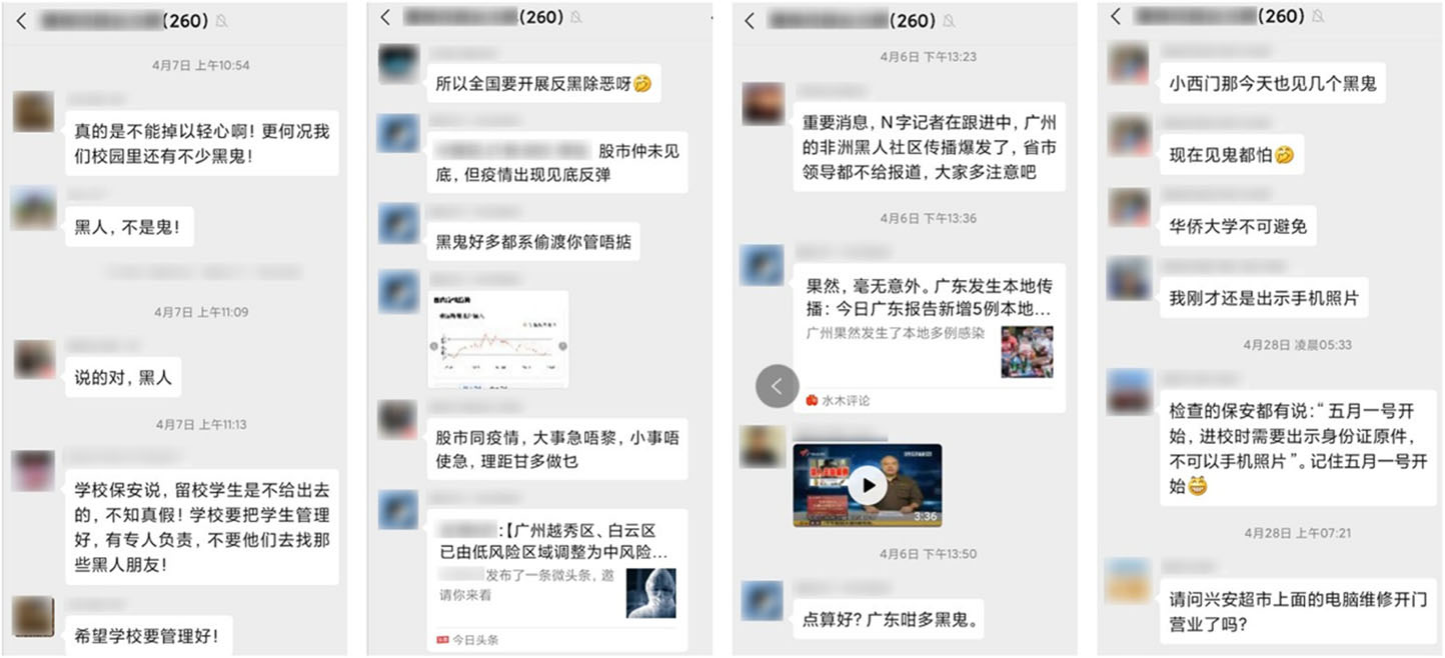
Discriminatory remarks about Africans in China in a WeChat group with 260 users based in Guangzhou. Africans were described as “*heigui*” (黑鬼, darkie/negro or black ghost/devil literally)

With the imported cases becoming the main source of new infections, the discrimination towards foreigners in China started to emerge despite the fact that most imported cases were returned Chinese citizens. The stigmatization of foreigners escalated after April 4 when a nurse in Guangzhou, a commercial hub as well as home to one of Asia’s largest populations of Africans, was violently attacked by an African man who refused to follow quarantine regulations. In addition, it was rumored that some communities in Guangzhou where Africans lived and traded were under lockdown after two Nigerians who had tested positive for the virus escaped. The rumor further led to Africans being condemned for transmitting the virus. As a result, hundreds of Africans were evicted from their rental apartments, turned down by hotels, and had to stay on the street for days (BBC April 17, 2020a). In addition, all Africans in Guangzhou were compulsorily tested for coronavirus while other non-African foreigners were not required to do so. Later on, the local authority reported that 111 out of 4500 Africans had tested positive for the coronavirus. Some local restaurants in Guangzhou refused the entry of Africans (Fig. 8), just as some mainland Chinese had been treated in Hong Kong (BBC May 20, 2020d). In other areas of China, Africans were also reported to have been harassed by local police and government officials, as well as being denied service by hospitals. In the cyberspace and social media, discriminatory and even hate-laden remarks to Africans were widely observed (Fig. 9).

The presence of stigma and severe discrimination against Africans in Guangzhou was widely reported by global mainstream media. It also triggered a diplomatic crisis between China and some African countries. Chinese ambassadors in many African countries were summoned by their hosts to complain about such racist and discriminatory treatment (BBC May 13, 2020c).

## Factors Affecting Stigma, Hate Incidents, and Crimes Amid Covid-19

**Fig. 10:**
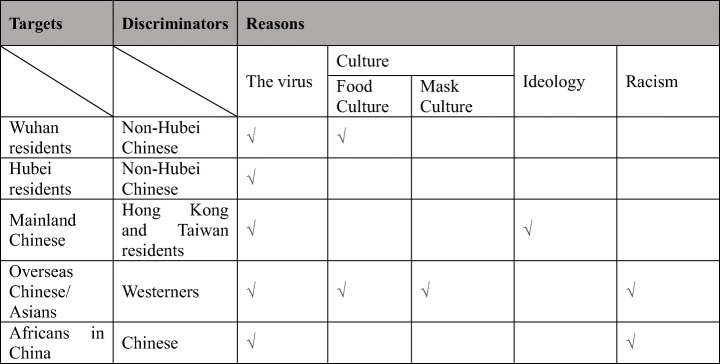
The corresponding factors for stigma to each targeted group

In the existing literature, several factors have been identified as shaping the bestowing of stigma on patients and the community in which the contagious diseases started. Besides the fear of diseases, media framing of the diseases, and government policy, social distance between stigmatizers and being stigmatized is also an important factor as those who are stigmatized tend to be outsiders (Wynne et al. 2014; Lee et al. 2005). Several themes emerged in this research as factors contributing to the stigma, hate incidents, and crimes experienced by targeted victim groups during the Covid-19 pandemic, including the fear of the virus, food and mask culture, political ideology, and racism. While fear of the virus played a central role in all cases, other factors played different roles to the targeted victim groups (Fig. 10).

### The Fear of Infection

The fear of getting infected was embedded in the stigma attached to all victim groups. As a highly contagious virus with uncertain effects and no cure available at the time, the outbreak of Covid-19 created great panic in society. In the early stage of the Covid-19 pandemic, it was widely reported that the coronavirus could survive in the air and on the surface of objects for hours and even days (Van Doremalen et al. 2020). Patients could, it was said, be infected by aerosol and fecal-oral transmission within seconds (Wang et al. 2020). A frequently circulated case was that one person had been infected after meeting a patient in the vegetable market for a mere 15 s (China News February 6, 2020b). People also worried about cluster infections like that which had occurred in Hong Kong’s Amoy Apartment in the 2003 SARS period in which over 300 residents got infected by the coronavirus through the sewage system (Bloomberg February 20, 2020). The dire situation in Wuhan, marked by a lack of medical supplies, a lack of medical personnel, and an increasing number of patients and deaths, all contributed to the public panic about the virus. In China and globally as well, there was a severe shortage of materials such as facial masks, goggles, and protection suits for medical personnel. All those items were largely beyond the reach of most ordinary citizens. In addition, there was no effective medicine to cure or prevent the virus (Chen et al. 2020; Xu et al. 2020c). The outcome was that the potential discreditable—those who were believed to have high risks of spreading the virus—were widely avoided and stigmatized and even become the victim of hate crimes.

### Food and Mask Culture

Culture served as a significant factor in influencing stigma (Somma et al. 2008). During Covid-19, different cultural understandings of the significance of food and mask significantly affected the discrimination extended towards targeted groups. The food culture of eating wild animals led to hostility against Wuhan citizens and overseas Chinese. Since the origin of the 2003 SARS epidemic was the horseshoe bat, and since the patients of the Covid-19 displayed similar symptoms to those of SARS patients, it was widely speculated that the virus may also have come from bats. The report that most early patients were from the Wuhan Southern China Seafood Market where wild animals were sold as food further strengthened this speculation. Residents from other provinces and cities started to condemn Wuhan residents for their eating practices. Curses such as “Wuhan residents who eat bats are damned!” were not uncommon in cyberspace. It was worth noting that before the outbreak of Covid-19, it was Cantonese rather than Wuhan residents who were widely known for eating wild animals in China. Nevertheless, when menus from the Southern China Seafood Market circulated and videos of drinking bat soup spread online, Wuhan residents become a target and were blamed for the outbreak of Covid-19.

Some overseas Chinese faced similar stigmata in their host countries. The representation of certain Chinese foods as primitive and dirty has long existed in western countries (Lu and Fine 1995). Such a stereotype was revived by the spread of the Covid-19. Overseas Chinese restaurants were affected by the revived stigma. It was reported that a Chinese restaurant in Canada was frequently harassed. Some provokingly called to buy bat soup while others asked the restaurant to close and threatened the safety of the shop owner (China News February 6, 2020b). It was reported that the stigma attached to Chinese food led to a sharp drop in sales at overseas Chinese restaurants. It was severe enough to trigger an intervention from local politicians. Paris mayor Anne Hidalgo made several visits to Chinese communities in early February to show her support. She also went to a Chinese restaurant in Paris Chinatown for lunch on February 1, 2020 (China News February 3, 2020a; CGTN February 6, 2020a). New York City officials, including the mayor De Blasio, visited Flushing’s Chinese community on February 13, and had lunch at a popular Chinese banquet hall while urging people not to panic or be discriminatory towards the consumption of Chinese food (CNN March 2, 2020e).

**Fig. 11:**
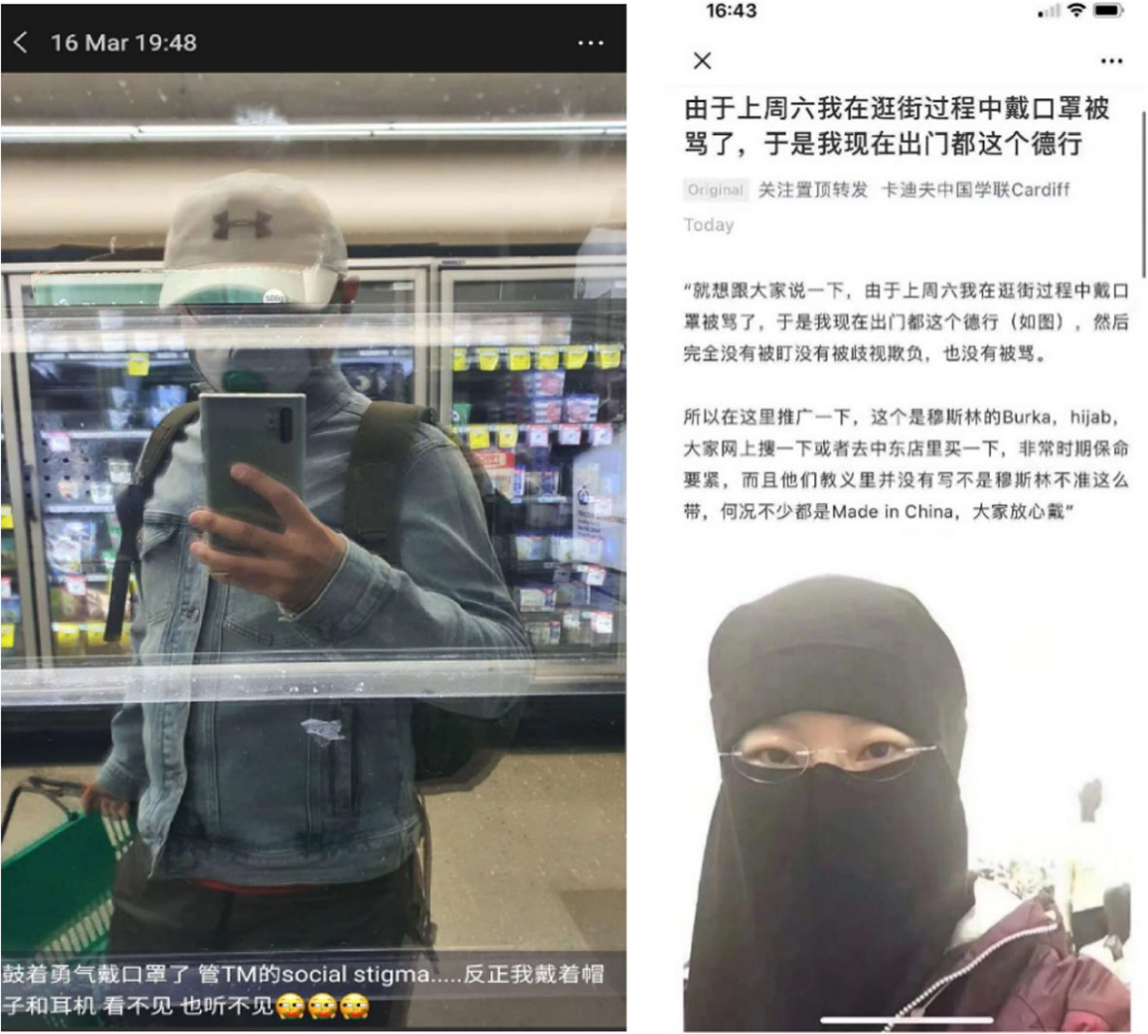
A Chinese overseas student in Australia posed a picture of his selfie with the comments that “I finally took up the courage to wear a mask. F**k social stigma. My cap and earphone will prevent me from seeing and hearing it (left).” A picture widely circulated in social media teaching people how to wear a Muslim Hijab to cover a facial mask inside to reduce stigma (right)

Besides food culture, different understandings of the wearing of masks also contributed to the vilification of overseas Chinese. In China and Asian societies, mask wearing is commonly practiced when people perceive there is a risk of infection. The 2003 SARS epidemic further popularized the wearing of masks. Others may also wear masks to cope with pollution. However, in many Western societies, mask wearing was not as popular as that in Asian societies. As Covid-19 spread all over the world, some overseas Chinese wore masks for precautionary reasons. However, some of those who wore them in public were regarded as spreading the virus or causing public panic. For instance, in the UK, the third worst-hit country outside the USA and Brazil with almost 45,000 fatalities by mid-July, mask-wearing was still not widely practiced (CNN July 15, 2020d). The US president Donald Trump was not seen wearing a mask in the public until July 12 (CNN July 12, 2020c). The stigma attached to the wearing of masks by overseas Chinese was so strong that some had to adopt creative ways of coping such as the wearing of caps and even the Muslim Hijab to hide their identities when going out (Fig. 11).

### Ideology

The tension between different political ideologies also contributed to the expression of discrimination against a number of groups. This was particularly the case when mainlanders were targeted by some Hong Kong and Taiwan residents.

Despite the liberalization of its economy, China remains an authoritarian regime politically (Xu et al. 2020a). Since Xi took power in 2012, China has become more conservative politically and assertive internationally. Its authoritarian nature was visible in how the Chinese government dealt with the pandemic in its early stage. As shown in Fig. 5, the first officially confirmed cases appeared during December 1–8, 2019, but the National Health Commission (NHC) did not inform the public until the last day of the month. In addition, the authorities tried to cover up the news and lied to the public. NHC repeatedly denied the possibility of human-to-human transmission, despite the fact that some medical workers were infected almost a month before Professor Zhong Nanshan finally reported this mode of transmission to the public. Eight whistle-blowers, including the most well-known, Dr. Li Wenliang, who later died from Covid-19 in early February, were disciplined by the police. Moreover, the local government did not take necessary measures to prevent the spread of the virus at an early stage. Large crowd gatherings such as the 10,000-crowd banquet in Baibuting neighborhood, a yearly celebrating events for Chinese New Year, were organized even in mid-January. By contrast, authorities in Hong Kong and Taiwan actively responded to the unknown illness at the very beginning, which posed a sharp contrast to the authoritarian handling of the case in mainland (Fig. 6). The discrimination against mainland Chinese in Hong Kong and Taiwan could also partially be attributed to the political tension between mainland China and Hong Kong and Taiwan.

**Fig. 12:**
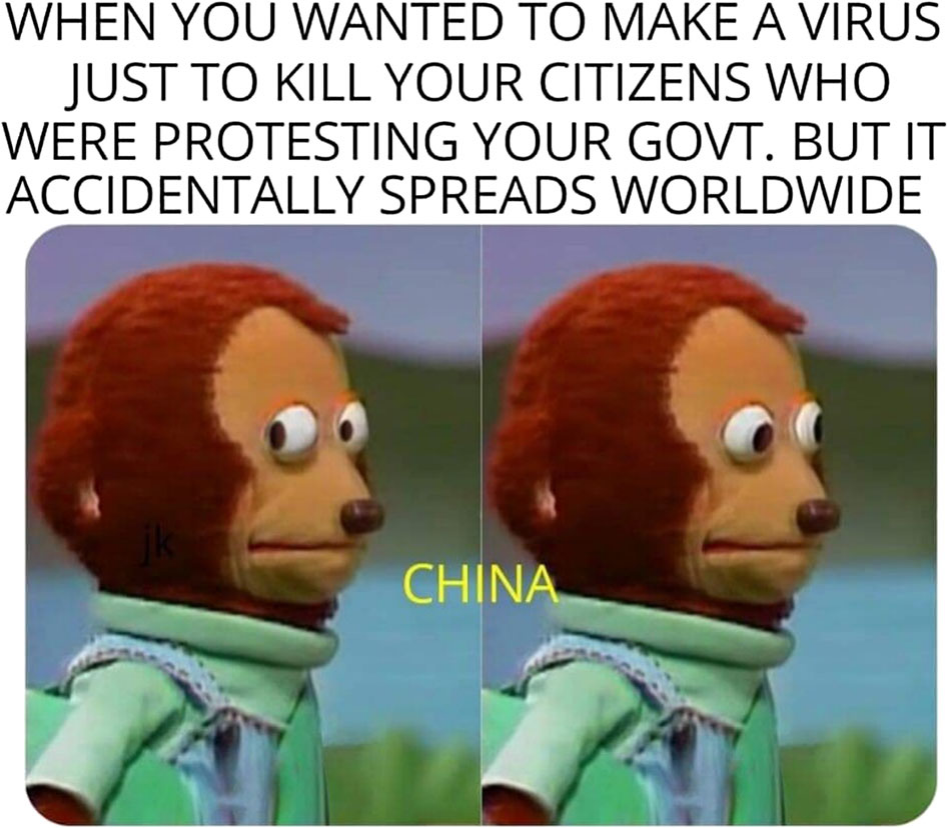
A widely circulated cartoon picture among Facebook users in Hong Kong about the rumor that coronavirus was human-made in China. In the cartoon, the monkey pretended to be innocent when his trick was revealed

The political tension between Hong Kong and mainland China became increasingly intensified after a long promised universal suffrage in Hong Kong was not realized. The 2014 Occupy Central Movement and 2019 anti-extradition bill protest further increased the political tension between Hong Kong and mainland China. The newly passed Hong Kong National Security Law in June 2020 was the latest demonstration of the problem. The outbreak of coronavirus became the latest battle field to be utilized in Hong Kong to criticize mainland China. For instance, a cartoon picture indicating that the coronavirus was man-made by the Chinese Communist Party (CCP) to kill dissidents but went awry was widely circulated in social media in Hong Kong (Fig. 12).

The impact of all this political tension on attitudes towards mainland Chinese was further revealed in some of the explicit hatred reporting in media. An article from *Apple Daily*, a pro-democracy newspaper in Hong Kong, wrote that:

“Over the past 70 years, not only did the CCP looked down upon the Chinese people, but most of the foreigners also disdained them. Even the Chinese people belittled themselves. Jackie Chan said that the Chinese need to be controlled. Yu Jie, a Chinese-turned-American stated that ‘China was the pronoun of ugliness, lowliness, cruelty, evil, and truculence. It was not my motherland’. Under the reign of the CCP, foreigners had no better choice but to look down upon Chinese people.” (Apple Daily February 25, 2020)

The political tension between Taiwan and mainland China has been similarly acute. This is particularly the case since the pro-independence Democratic Progressive Party (DPP) took power. In Taiwan, the quarantine measures were regarded as discriminatory against mainland Chinese when the authority strictly regulated the entry of the children of Taiwanese-mainlanders but did not put all Western arrivals under quarantine when the situation in many Western countries had worsened in April (Taiwan Net April 21, 2020).

**Fig. 13:**
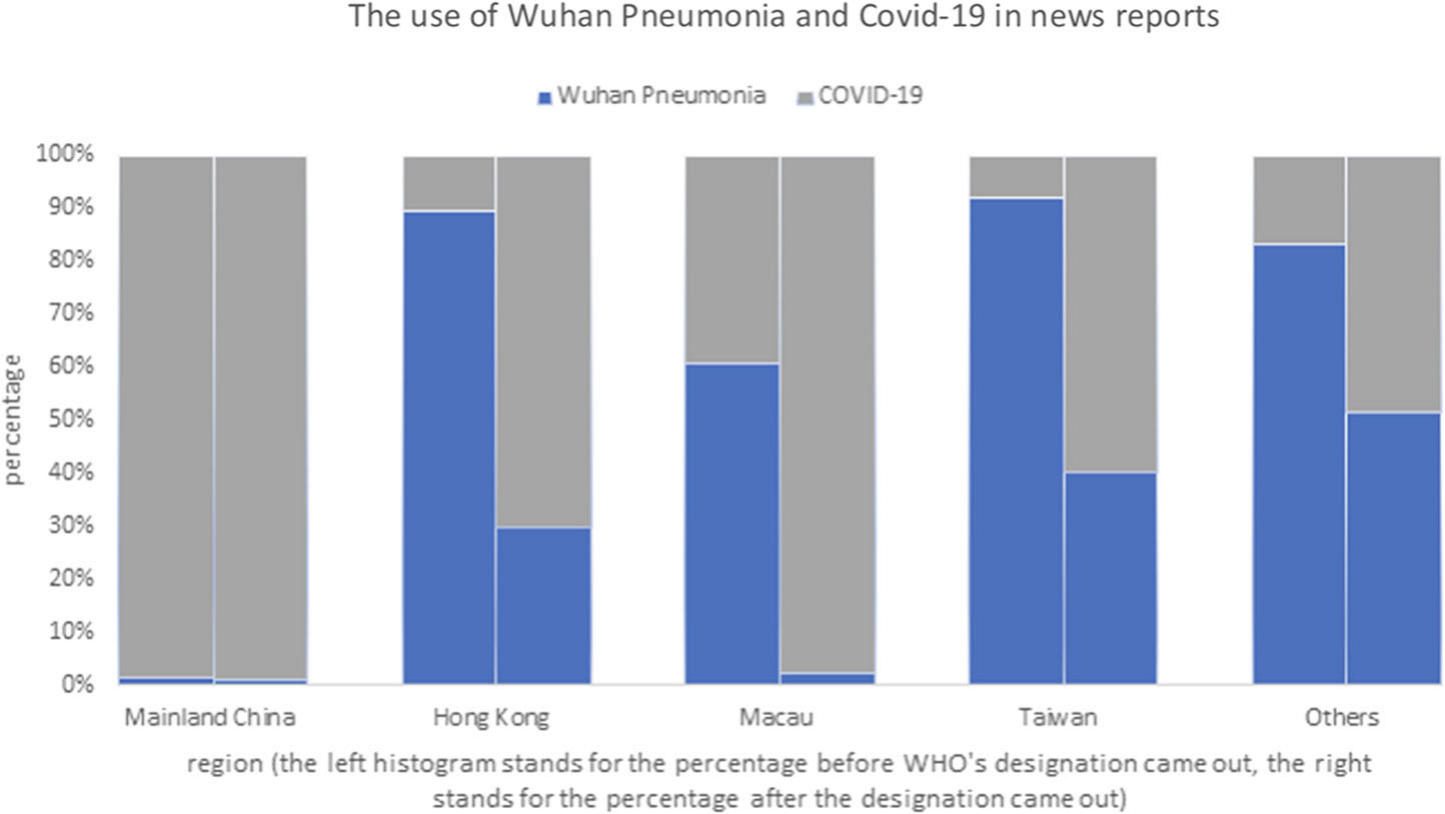
The use of “Wuhan pneumonia” and “Covid-19” in news reports in mainland China, Hong Kong, Macau, Taiwan, and other countries before and after WHO renaming the pandemic as “Covid-19” on February 11, 2020. (26,203 news were collected from December 8, 2019, to February11, 2020, and 74,149 news were collected during February 11 to March 11, 2020)

The stigma adhering to mainland Chinese could also be seen in how the media in Hong Kong and Taiwan used the term “Wuhan pneumonia.” At the early stage of the pandemic, “Wuhan pneumonia” was widely used to refer to the disease. WHO renamed the disease as Covid-19 on Feb 11, 2020, to avoid a stigmatizing allusion to Wuhan. However, the discriminatory term was continuously widely used in Hong Kong and Taiwan. The data from our content analysis of newspaper articles showed that before WHO’s renaming of the pandemic, nearly 90% of reports used the term “Wuhan pneumonia” in Hong Kong, Taiwan, and overseas Chinese language newspapers. After the renaming, around 30, 40, and 50 percentages of reports still used the term. On the other hand, newspapers in Macau, another SAR, nearly completely abandoned using the discriminatory term (Fig. 13), a reflection of its closer relationship to mainland China (Xu et al. 2020b).

### Racism

Rooted racism is an important factor causing hate incidents and crimes to overseas Chinese and Asians as well as the stigma faced by Africans in China. The racism experienced by overseas Chinese is nothing new. Those living and working in the USA used to be regarded as dirty and uncivilized and were treated as “yellow peril” during the first wave of Chinese immigrants to the USA in nineteenth century (Mayer 2014). The discriminatory term “Chink” was widely used to refer to them. The Chinese Exclusion Act passed in 1882 further formalized the racism addressed to Chinese people and the discriminatory policy lasted until mid-twentieth century (HuffPost April 28, 2020). Despite the fact that Asians are now the fastest growing population and often labeled as a “model minority” who enjoy considerable success in the USA (Lee 2009), Covid-19 became the new trigger to revive a deeply rooted discrimination against Chinese and Asians in general. The racist nature of discrimination towards overseas Chinese and Asians could also be reflected in the headlines of mainstream global media, such as “Why don’t you stay home? Coronavirus sparks racism fears” (Financial Times February 1, 2020), “North Yorkshire Police probe racist coronavirus-related incidents” (BBC February 6, 2020b), “FBI warns of potential surge in hate crimes against Asian Americans amid coronavirus” (ABC March 27, 2020), “Covid-19 has inflamed racism against Asian-Americans. Here’s how to fight back” (CNN April 11, 2020b), and “The slur I never expected to hear in 2020” (NYTimes April 12, 2020). The US president Donald Trump’s insistence in using “Chinese Virus” further fueled racial discrimination towards Chinese. A piece of research found that after Trump’s use of the epithet “Chinese virus,” it increased tenfold in Twitter in the USA in the following week (Budhwani and Sun 2020).

Racism also played its role of discrimination faced by Africans in China. There is evidence to point to a considerable level of racism towards Africans in China (Zhou et al. 2016). While the white population are perceived to occupy a higher rank in the hierarchy of racial stratification, Africans were often look down upon in China (Williams 2011). One recent example was an advertisement for a Chinese washing detergent in which a black man was pushed into the washing machine to be “washed” into becoming a lighter-skinned Asian (The Guardian May 28, 2016). When China faced the second wave of the outbreak due to imported cases in April, all Africans in Guangzhou were compulsorily tested and some of them were repeatedly tested, while many non-African foreigners were not required to comply (BBC May 20, 2020d). During the outbreak, racial discriminatory terms such as “*heigui*” (黑鬼, darkie/negro or black ghost/devil literally) were commonly used in Chinese social media to refer to Africans. While some Africans were evicted by landlords and had to stay on the street, others were widely avoided by local residents. Indeed, racism never seems to disappear but adapts to new circumstance. The outbreak of the pandemic simply allowed racism and xenophobia to revive (Devakumar et al. 2020).

## Conclusion

Hate crimes have been an important sub-branch in criminology. Victims of hate crimes may be attacked by hostility or prejudice based on their disability, race, religion, sexual orientation, or gender identity (Perry 2001; Jacobs and Potter 1998). Amid the Covid-19 pandemic, various groups of population became victims of stigma and even hate crimes. In this study, using data collected from semi-structured interviews, online observation, and media content analysis, we explore how Covid-19 was associated with the occurrence of stigma, discrimination, and hate crimes towards various victim groups in the Chinese-speaking world.

We found that along with the spread of Covid-19, Wuhan residents, Hubei residents, mainland Chinese, overseas Chinese/Asians, and Africans in China became the targets of stigma, discrimination, and even hate crimes one after another. We further explored what factors contributed to the victimization. It was found that while fear of infection was the common basis for all targeted groups, the food culture of eating wild animals affected stigmata towards Wuhan residents and the stereotype of Chinese food culture affected stigma towards oversea Chinese. In addition, we found that different understanding of mask-wearing affected stigma towards oversea Chinese, and the political ideology contributed to the stigmata onto mainland Chinese in Hong Kong and Taiwan. Lastly, the revived racism shaped the stigmatization to both oversea Chinese in Western countries and Africans in China.

This research documents how and why Covid-19 caused stigma and hate crimes in the Chinese-speaking world. A better understanding of the nature of disease-related stigma and hate crimes may provide a basis for policy-makers to address these problems in future. Future research can address an important but not fully discussed factor for the stigma and discrimination amid the Covid-19: the social distance. Sociologists have long revealed that people tend to think danger and pollution always come from elsewhere, from outside, not from “us” (Schutz 1976; Douglas 1966). During the Covid-19 outbreak, the source of the infection was over and over again believed from there, not from here, from without, and what was without depended in large measure on scale. Correspondingly, the size of the stigmatized group considered responsible for the infection grew both in numbers and in generality, from Wuhan citizens, to Hubei citizens, to mainland Chinese, to all Chinese and finally people of vaguely Asiatic appearance. An exploration of how social distancing contributed to the stigma and hate crimes in the Covid-19 pandemic will further provide new knowledge in how to prevent them from happening in future.

## Footnotes

1 See a map of Hubei province and Wuhan city in Fig. 2

2 The team included the first author and 6 undergraduate student co-authors who took the his course in Qualitative Research Methods at the University of Macau when the pandemic started in early 2020.
